# Past distribution of epiphyllous liverworts in China: The usability of historical data

**DOI:** 10.1002/ece3.4274

**Published:** 2018-07-02

**Authors:** Yanbin Jiang, Tiejun Wang, Yupeng Wu, Ronggui Hu, Ke Huang, Xiaoming Shao

**Affiliations:** ^1^ Key Laboratory of Arable Land Conservation (Middle and Lower Reaches of Yangtze River) Ministry of Agriculture College of Resources and Environment Huazhong Agricultural University Wuhan China; ^2^ Faculty of Geo‐Information Science and Earth Observation (ITC) University of Twente Enschede The Netherlands; ^3^ Key Laboratory of Ecosystem Network Observation and Modeling Institute of Geographic Sciences and Natural Resources Research Chinese Academy of Sciences Beijing China; ^4^ Beijing Key Laboratory of Biodiversity and Organic Farming College of Resources and Environmental Sciences China Agricultural University Beijing China

**Keywords:** bryophytes, environmental variables, historical records, maxent, species distribution model

## Abstract

Epiphyllous liverworts form a special group of bryophytes that primarily grow on the leaves of understory vascular plants in tropical and subtropical evergreen broadleaf forests. Being sensitive to moisture and temperature changes, epiphyllous liverworts are often considered to be good indicators of climate change and forest degradation. However, they are a poorly collected and taxonomically complicated group, with an only partly identified distribution pattern. In this study, we built four models based on 24 environmental variables at four different spatial resolutions (i.e., 1 km, 5 km, 10 km, and 15 km) to predict the past distribution of epiphyllous liverworts in China, using Maxent model and 63 historical location records (i.e., presence‐only data). Both area under the curve of the receiver operating characteristic (AUC) and true skill statistic (TSS) methods are used to assess the model performance. Results showed that the model with the predictors at a 15‐km resolution achieved the highest predictive accuracy (AUC=0.946; TSS=0.880), although there was no statistically significant difference between the four models (*p *>* *0.05). The most significant environmental variables included aridity, annual precipitation, precipitation of wettest month, precipitation of wettest quarter, and precipitation of warmest quarter, annual mean NDVI, and minimum NDVI. The predicted suitable areas for epiphyllous liverworts were mainly located in the south of Yangtze River and seldom exceed 35°N, which were consistent with the museum and herbarium records, as well as the historical records in scientific literatures. Our study further demonstrated the value of historical data to ecological and evolutionary studies.

## 1 INTRODUCTION

**Figure 1:**
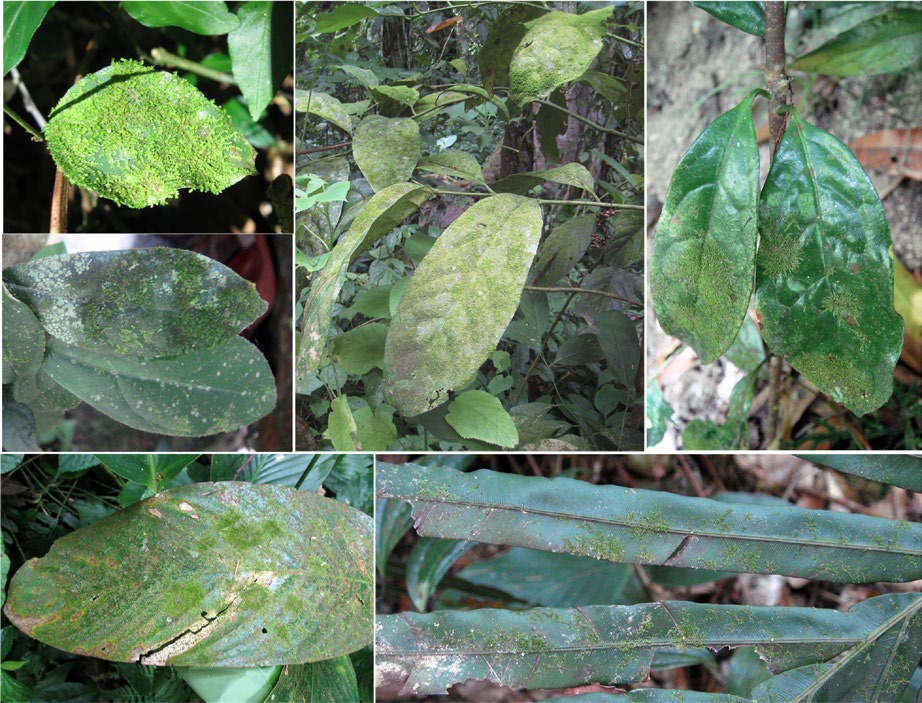
Epiphyllous liverworts growing on leaves of various vascular plants. Photographs by Yanbin Jiang

Epiphyllous liverworts, a special group of bryophytes that grow on the leaves of understory vascular plants, often inhabit constantly moist and warm forests in tropical and subtropical regions (Chen & Wu, 1964; Figure 1). There are three types of epiphyllous liverworts: obligate, facultative, and occasional. The obligate epiphyllous liverworts occur exclusively on living leaves. The facultative epiphyllous liverworts occur predominantly on living leaves but can grow on other substrates. While the occasional epiphyllous liverworts seldom occur on living leaves, but predominantly present on other substrates. Both obligate and facultative species belong to typical epiphyllous liverworts (Zhu & So, 2001). They are particularly sensitive to moisture and temperature changes and are regarded as potential indicators of climate change and forest degradation or integrity (Jiang et al., 2014; Pócs, 1996). Epiphyllous liverworts have been mainly found in Asia, Australia, Africa, Central and South America, and Macaronesian islands in Europe at latitudes of about 30 degrees north and south of the equator. At times, they have been found in regions at much higher latitudes such as Madeira (32.5°N) (Sjögren, 1975) and the Azores (38.5°N) (Sjögren, 1997) in Portugal, the Appalachians (35.0°–37.97°N) (Davison, 1997; Risk, Richardson, & Davison, 2011; Schuster, 1959), Caucasus Mountains (43.5°N) in Russia (Pócs, 1982), Sikoku (33.75°N) (Kamimura, 1939) and Niigata Prefecture (38°N) (Shirasaki, 1997) in Japan, Chiltern Hills (51.75°N) in Britain (Porley, 1996), and British Columbia (49.42°N) in Canada (Vitt, Ostafichuk, & Brodo, 1973).

In China, field surveys and studies on epiphyllous liverwort have been conducted for almost a century (Chen & Wu, 1964). Approximately 168 epiphyllous liverwort species have been found in China due to its diverse topography and climatic conditions, with a relatively high endemism rate and high conservation status (Zhu & So, 2001). These species are widely distributed in tropical rainforests and subtropical evergreen broad‐leaved forests throughout the Chinese provinces within 30 degrees north latitude, including Anhui, Fujian, Guangdong, Guangxi, Guizhou, Hainan, Hongkong, Hubei, Hunan, Jiangxi, Sichuan, Taiwan, Tibet, Yunnan, and Zhejiang (Chen & Wu, 1964). However, recent studies also found them in regions even further north (i.e., 31°N), including Guanxian county in Sichuan province (Luo, 1990) and Houhe Nature Reserve in Hubei province (Peng, Liu, & Wu, 2002). The spatial distribution of epiphyllous liverworts may vary over time because of changes in climate and habitat conditions.

The temporal patterns of species distribution can be examined by drawing a biological inference from species locational data of various periods via a GIS‐based species distribution model (Butcher et al., 2014; Guisan & Thuiller, 2005; Guisan & Zimmermann, 2000). Species distribution models (SDMs) are widely used in ecology and conservation, which relate species occurrence data to environmental predictor variables on the basis of statistically or theoretically derived response surfaces (Guisan & Zimmermann, 2000). Species occurrence data can be categorized as simple presence or presence–absence observations based on random or stratified field sampling or records obtained from natural history collections (Graham, Ferrier, Huettman, Moritz, & Peterson, 2004). Environmental variables can directly or indirectly affect species. Biologists have long been attempting to identify where a species will be in the future and to predict its temporal and spatial distribution in unknown regions on the basis of geographical distribution data of species in the past and present (Moya, Jacome, & Yoo, 2017; Ning, Wei, & Feng, 2017). Understanding the spatial dynamics of species over time and their driving factors has a critical role in resource utilities, potential risk assessment, and conservation planning (Guisan & Thuiller, 2005; Johnson, Ober, & Adams, 2017). SDMs have already been applied for predicting the current distribution of epiphyllous liverworts (Jiang et al., 2014). However, the spatial and temporal dynamics of epiphyllous liverworts remain unknown. Epiphyllous liverworts are likely to be among the groups of organisms that would benefit most strongly from the use of historical records for ecological and conservation research, because these species have fast generation times and tightly coupled with the local environment. Therefore, natural history collections housed in museums and herbaria, as well as bibliographic records of historical data, may be used to predict their distribution and change.

A significant limitation of historical records of epiphyllous liverworts is the uncertainty about where the occurrences are located. The local name, altitude, habitat, and collection time are the only valuable information available in most of the presence data. How historical records can be used to characterize the propagation patterns of epiphyllous liverworts, therefore, needs to be determined to examine the distribution range at the regional scale. A set of predictors available at fine resolution (grain size) may also need to be aggregated to coarser resolutions (Guisan, Graham, Elith, & Huettmann, 2007). Thus, this study aims to examine the past distribution of epiphyllous liverworts in China based on historical records of epiphyllous liverworts as well as environmental variables at different spatial resolutions. In particular, we set out to address the following questions: (a) How do spatial resolution (grain size) changes affect model performance using historical records for modeling the distribution of epiphyllous liverworts? (b) How wide is the modeled distribution of epiphyllous liverworts across China under the different spatial resolutions? (c) Which abiotic or biotic factors (e.g., topography, temperature, precipitation, and vegetation) limit the geographical distribution of epiphyllous liverworts at various spatial resolutions (e.g., 1 km, 5 km, 10 km, and 15 km) ?

## 2 METHODS

### 2.1 Species data

**Figure 2:**
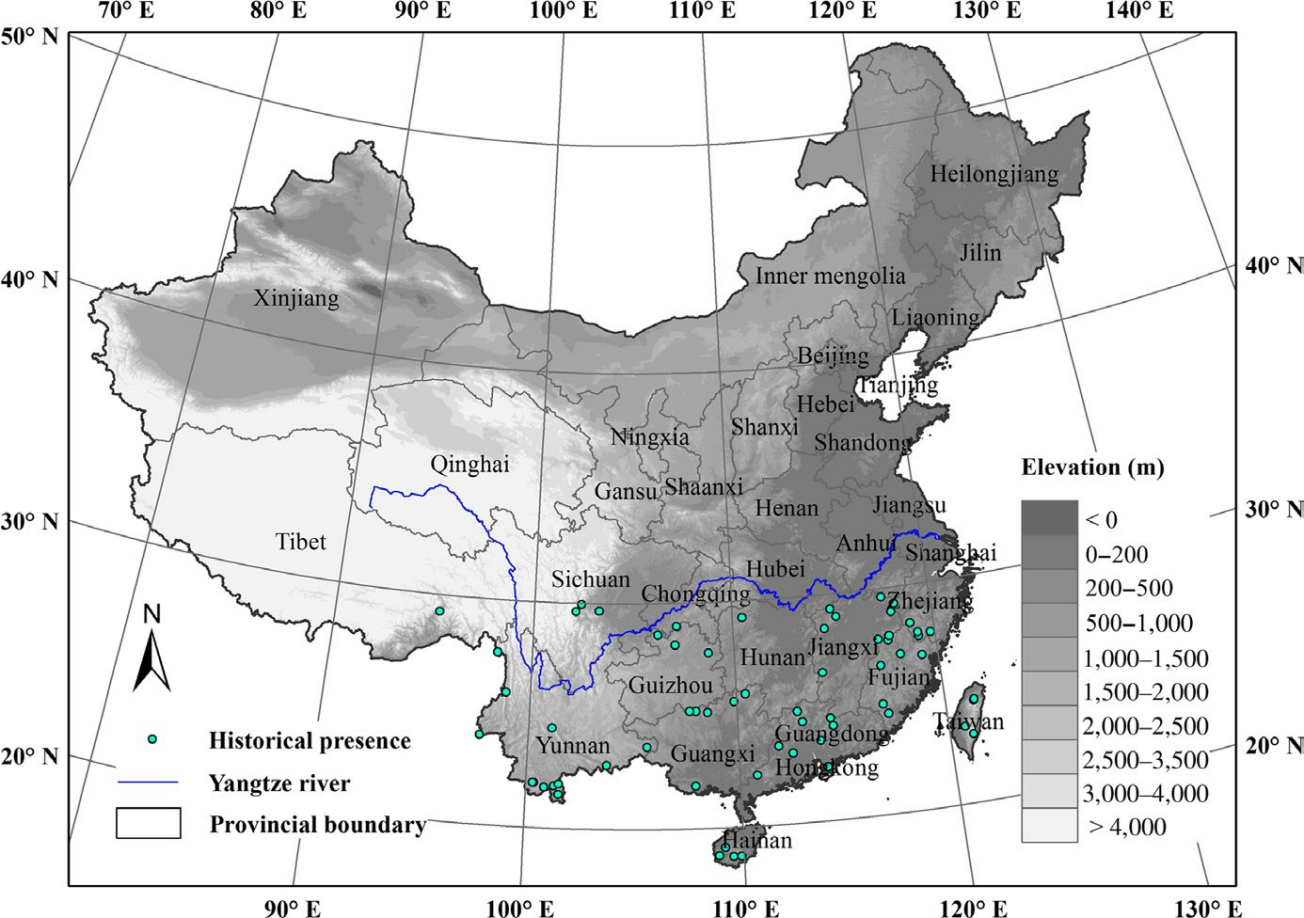
Study area and locations of the 63 occurrence records of epiphyllous liverworts in China used in the species distribution models

Typical epiphyllous liverworts, including both obligate and facultative species, were considered as the target species of the current study. In total, about 140 epiphyllous liverworts species belonging 28 genera of 11 families were involved. We considered all these species as a “species group.” These species occurrence data were composed of historical records collected before 2000 (Appendix 1). These records were derived from publications (1964–2001) and natural history collections from the Herbarium, Institute of Botany, Chinese Academy of Sciences (1954–1994). Most of the location information of the historical species data was presented as descriptions of localities with variable accuracy. Records with the vague location information, such as province, county, or locations that cannot be found, were excluded in this study. We approximated geo‐referenced point localities through Google Earth and the Vegetation Map of China (1:4,000,000) (http://westdc.westgis.ac.cn), considering the following three factors: (a) local name; (b) elevation; and (c) forest distribution. The geo‐referenced historical data may have variable location accuracy, while accurate occurrence records are available at high resolutions (Engler, Guisan, & Rechsteiner, 2004). Occurrence localities with a distance of at least 15 km were retained to lessen spatially autocorrelated effect. A total of 63 historical records with the estimated location in the range of the study area were obtained and plotted in Figure 2.

### 2.2 Environmental variables

Three categories of environmental variables, including bioclimatic data, topographic data, and satellite‐derived vegetation indices, were used to predict the epiphyllous liverworts distribution in this study.

We downloaded 19 bioclimatic variables from the WorldClim website (http://www.worldclim.org/). WorldClim is a set of global climate layers (climate grids) at a 1‐km resolution, which was generated by interpolating observations from over 4,000 weather stations around the world between 1950 and 2000 (Hijmans et al., 2005). We also downloaded the potential evapotranspiration (PET) and Aridity Index (AI) datasets from the CGIAR‐CSI GeoPortal (http://csi.cgiar.org). Both PET and AI grid layers are available at 1‐km spatial resolution representing the annual average over the 1950–2000 period. PET is a measure of the ability of the atmosphere to remove water through evapotranspiration processes. AI defined as the ratio of annual potential evapotranspiration to annual precipitation, which can be used to quantify precipitation availability over atmospheric water. AI values increase for more humid conditions and decrease with more arid conditions.

Topography is a relatively static variable compared with other biophysical factors, including climate, functioning as a key driver of biodiversity (Rosenzweig, 1995). We downloaded the GTOPO30 digital elevation model (DEM) data from the U.S. Geological Survey website (https://lta.cr.usgs.gov/GTOPO30), which has a 30‐arc‐seconds (approximately 1 km) spatial resolution. Then, we generated slope and aspect data layers from the GTOPO30 DEM using ArcGIS 10.1.

Satellite‐derived Normalized Difference Vegetation Index (NDVI) data contributed significantly to the distribution of epiphyllous liverworts (Jiang et al., 2014). As the species occurrence data were derived from 1936 to 1999 (Appendix 1) and the climatic data were derived from 1950 and 2000, the only time‐equivalent NDVI data source was the GIMMS NDVI (http://glcf.umiacs.umd.edu/data/gimms/). The GIMMS NDVI is originated from 1981, with a resolution of ~8 km. A time series of 20 yearly (1981 to 2000) averaged images was generated and used to calculate meaningful NDVI indices: annual maximum NDVI, annual mean NDVI, annual minimum NDVI, and NDVI standard deviation.

All the environmental variables were firstly resampled and projected as GIS raster layers in GCS_WGS_1984 at ca. 1‐km resolution. Then, the 1‐km variables were aggregated to coarser resolutions of 5 km, 10 km, and 15 km, and converted all layers to ASCII format for use in Maxent. Projection and aggregation were implemented in ArcGIS 10.1. Table 1 shows the details of all the environmental variables used for modeling.

**Table 1:** Environmental variables used for modeling the distribution of epiphyllous liverworts

Data source	Category	Variables	Abbreviation	Units
WorldClim	Bioclimatic	Annual Mean Temperature	Bio1	^o^C × 10
		Mean Diurnal Range (Mean of monthly (max temp ‐ min temp))	Bio2	^o^C × 10
		Isothermality (BIO2/BIO7) (* 100)	Bio3	%
		Temperature Seasonality (standard deviation *100)	Bio4	^o^C × 10
		Max Temperature of Warmest Month	Bio5	^o^C × 10
		Min Temperature of Coldest Month	Bio6	^o^C × 10
		Temperature Annual Range (BIO5‐BIO6)	Bio7	^o^C × 10
		Mean Temperature of Wettest Quarter	Bio8	^o^C × 10
		Mean Temperature of Driest Quarter	Bio9	^o^C × 10
		Mean Temperature of Warmest Quarter	Bio10	^o^C × 10
		Mean Temperature of Coldest Quarter	Bio11	^o^C × 10
		Annual Precipitation	Bio12	mm
		Precipitation of Wettest Month	Bio13	mm
		Precipitation of Driest Month	Bio14	mm
		Precipitation Seasonality (Coefficient of Variation)	Bio15	%
		Precipitation of Wettest Quarter	Bio16	mm
		Precipitation of Driest Quarter	Bio17	mm
		Precipitation of Warmest Quarter	Bio18	mm
		Precipitation of Coldest Quarter	Bio19	mm
CGIAR‐CSI	Bioclimatic	Potential Evapotranspiration	PET	mm
		Aridity index	AI	/
USGS GTOPO30	Topographic	Altitude	Altitude	m
		Aspect	Aspect	degree
		Slope	Slope	degree
GIMMS	Vegetation	Annual minimum NDVI	NDVI_min	/
		Annual mean NDVI	NDVI_mean	/
		Annual maximum NDVI	NDVI_max	/
		Standard deviation NDVI	NDVI_std	/

### 2.3 Species distribution modeling

Maximum entropy (Maxent) modeling is a general‐purpose method for characterizing probability distributions from incomplete information (Phillips, Anderson, & Schapire, 2006). The Maxent method does not require absence data, making it appropriate for modeling species distributions based on presence‐only historical species records. We used Maxent software (version 3.3.3e, http://www.cs.princeton.edu/~schapire/maxent/) to generate the species distribution model. Recommended default values of convergence threshold (10^−5^) and maximum number of iterations (500) were used when building the model. We generated 10,000 random points (i.e., background or pseudo‐absence sample points) from the whole study area. Suitable features and regularization values used can reduce model overfitting and complexity (Warren & Seifert, 2011). According to Phillips and Dudik (2008), combinations of features including linear (L), quadratic (Q), and hinge (H) were set by default in Maxent when species occurrence samples were 15 to 79. We thus practiced the L, LQ, H, and LQH features, with regularizations of 0.5, 1, 1.5, 2, 2.5, 3, 3.5, and 4, respectively, in order to select the optimal settings of features and regularization. The selection of features and regularization was carried out based on the sample size corrected Akaike information criteria (AICc) (Warren & Seifert, 2011). The default logistic output of Maxent is continuous variables ranging from 0 to 1, where high values indicate high relative suitability.

### 2.4 Model scenarios, evaluation, and statistical analysis

To determine the proper resolution of accurately modeling the past distribution of epiphyllous liverworts, we developed four model scenarios using the same species dataset (63 historical records). The spatial resolution of these environmental layers was at 1 km, 5 km, 10 km, and 15 km, and each level of layers together with species dataset was a model scenario. To avoid sampling bias of species occurrence data, we used a bias file in each model scenario. The bias file was generated based on the point localities of historical records by applying kernel density function (Elith, Kearney, & Phillips, 2010).

To facilitate model evaluation, we used the cross‐validation approach with 10 replicates in the Maxent for each model scenario. During the cross‐validation, the species dataset was divided into 10 random partitions, and the model was operated 10 times with each of the 10 partitions as a testing set (six or seven occurrence localities); the other nine partitions were used as a training set (57 or 56 occurrence localities) in a replicate. As a result, 10 datasets, including predicted values of training and testing localities and 10,000 background (pseudo‐absence) localities, were generated automatically. We integrated 10 replicates for model evaluation, with a testing prediction and a corresponding background prediction by each replicate. Each locality in an evaluation replicate has two values: One is the observed occurrence value (background points = 0; test presence points = 1), and the other is the predicted value derived from the logistic output of the Maxent model.

To evaluate the predictive accuracy of models, we used both threshold‐independent and threshold‐dependent methods. The area under the curve (AUC) of the receiver operating characteristic (ROC) is a dominant tool in evaluating the accuracy of models predicting distributions of species because the ROC has the advantage of being threshold‐independent. The resulting AUCs range from 0 to 1, with 1 indicating a perfect fit of the model, > 0.9 signifying excellent model performance, 0.7–0.9 as moderately useful models, < 0.7 for poor model performance, and “0.5” indicating randomness (Pearce & Ferrier, 2000). Considering that AUC cannot be used as a standard and sufficient measurement of accuracy in species distribution models (Austin, 2007), we also used the true skill statistic (TSS), a threshold‐dependent method. TSS is calculated by adding sensitivity and specificity together and subtracting 1. The TSS values range from −1 to 1, and 1 indicates a perfect fit and values of 0 or less indicate a performance no better than the random model (Allouche, Tsoar, & Kadmon, 2006). AUC and TSS were calculated for this 10‐fold of evaluation dataset and averaged. Considering that the TSS values were calculated for all possible thresholds ranging from 0 to 1, only the maximum TSS (TSS_max_) value for species prediction was reported. To measure the effect of resolution on model performance, we compared the average AUC and TSS of each resolution through one‐way ANOVA. We also calculated Akaike information criterion (AIC_c)_ values for all model scenarios to evaluate the suitability of model selection. The model selection and evaluation statistics were carried out using the “ENMeval” and “PresenceAbsence” packages in R v 3.4.4 (R Development Core Team 2017).

To assist model interpretation, each model scenario was also operated on the full set of occurrence localities, taking advantage of all available data to provide the optimal estimates of the potential species distribution and the relative importance of the environmental variables. Assessing the best cutoff value for discriminating estimated presence and absence is usually ideal. Several approaches have frequently been used to determine optimal threshold. The lowest predicted value was associated with any one of the observed presence records, named lowest presence threshold (LPT) (Jackson & Robertson, 2011; Pearson, Raxworthy, Nakamura, & Peterson, 2007; Saatchi, Buermann, Ter Steege, Mori, & Smith, 2008; Sérgio, Figueira, Draper, Menezes, & Sousa, 2007). The fixed thresholds that reject only the lowest 10% of possible predicted values (T10) were then examined (Pearson et al., 2007). The additional one is the value that corresponds to the point on the ROC curve where sensitivity and specificity are maximized (Max Sensitivity + Specificity) (Braunisch & Suchant, 2010). We selected the second one because the thresholds of four model scenarios from the LPT were small and the maximum Sensitivity + Specificity significantly differed (Appendix 2). On the basis of the determined thresholds, we compared the spatial distribution range of epiphyllous liverworts at four spatial resolution levels. We applied the Jackknife test to diagnose which environmental variables were the key predictor variables to create the models (Prates‐Clark, Saatchi, & Agosti, 2008). The importance of an environmental variable is determined on the basis of obtaining a large training gain when the variable is used alone in the model and a subsequent decrease in training gain when removed from the model. The response curves were also plotted to demonstrate how variables affected the presence probability of epiphyllous liverworts being present. The response curves used all point localities and the respective environmental variable in isolation, and, thus, do not include interactions with other environmental variables (Phillips et al., 2006).

## 3 RESULTS

### 3.1 Model performance

According to AIC_c_ criteria, models with LQH features and regularization of 0.5 were selected. For all model scenarios, the AUC values were significantly higher than those of the random model (*p = *0.000). The high AUC (all > 0.9) and TSS_max_ (all > 0.7) values implied a robust performance of the Maxent model in capturing the variation in environmental variables over historical presence localities of epiphyllous liverworts. Coarsened resolution trends exhibited insignificant degradation or improvement of model performance according to AUC and TSS (*p *>* *0.05, one‐way ANOVA). The 15‐km resolution models obtained the highest AUC and maximum TSS when compared with the three other models. By assessing the AIC_c_ values, the 15‐km resolution model also exhibited the best performance with the lowest AIC_c_ (Table 2).

**Table 2:** Performance of models in predicting the distribution of epiphyllous liverworts at 1‐km, 5‐km, 10‐km, and 15‐km resolutions, showing threshold‐independent and threshold‐dependent model evaluation results by AUC and maximum TSS (TSS_max_) in *R* (10,000 background points used as pseudo‐absence for AUC and TSS_max_)

Model	AUC	TSS_max_	AICc
1 km	0.926 ± 0.062	0.760 ± 0.155	1840.243 ± 12.401
5 km	0.936 ± 0.029	0.834 ± 0.075	1472.627 ± 4.320
10 km	0.932 ± 0.039	0.740 ± 0201	1291.717 ± 4.682
15 km	0.946 ± 0.027	0.880 ± 0.011	1173.088 ± 5.283
*p*‐value	0.750	0.082	0.000

*Note*

One‐way ANOVA was performed to assess the effect of spatial resolution on model performance.

### 3.2 Comparison of predictive performance

**Figure 3:**
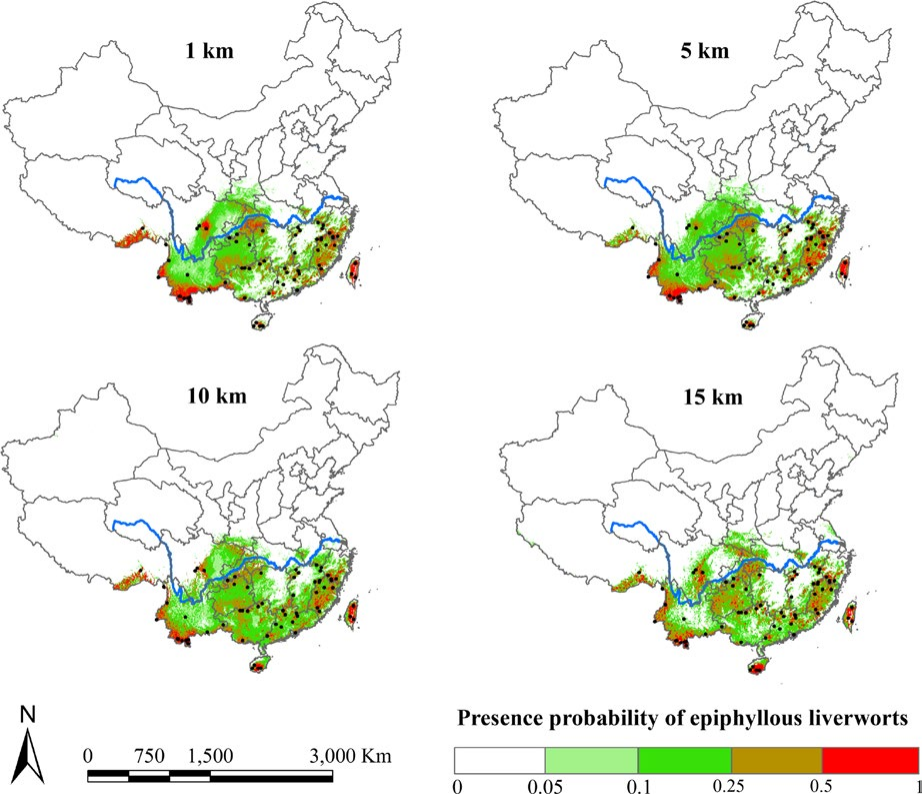
Maps showing the spatial distribution pattern of epiphyllous liverworts in China from four different model scenarios of 1 km, 5 km, 10 km, and 15 km resolutions

We derived four distribution maps of epiphyllous liverworts over entire China from four model scenarios on the basis of the environmental variables at 1 km, 5 km, 10 km, and 15 km resolutions, respectively. Logistic presence probability of epiphyllous liverworts is depicted in Figure 3. They exhibited similar distribution range; the north distribution extension did not exceed 35°N, and the most likely occurrence area was located in the south of Yangtze River. The variability between predictions by visual inspection, however, demonstrated that the distribution pattern of epiphyllous liverworts was yet influenced by resolution, and the probabilities spatially differed at various resolutions. Along the coarsening of resolution, the distribution patches of epiphyllous liverworts were more fragmented, and the high suitable area (presence probability > 0.5) was decreasing. To facilitate comparison of the visual output maps, a threshold that rejected the lowest 10% of training presence was used to indicate the probability of presence or absence, as shown in Table 3. At this threshold, the fractional predicted area demonstrated the fraction of all pixels predicted suitable for the species. The overall predicted area was low, with an average of 7.3% area of entire China, indicating that epiphyllous liverworts only occur in a limited range in China. The 1‐km resolution model obtained the highest predicted area (8.1%), while the 15‐km resolution model had the lowest (6%), with the AUC and TSS rankings reversed.

**Table 3:** Threshold for determining epiphyllous liverwort presence and corresponding fractional predicted area identified as presence for each model

Model	Logistic threshold	Fractional predicted area	*p*‐value
1 km	0.210	0.081	<0.001
5 km	0.242	0.073	<0.001
10 km	0.206	0.080	<0.001
15 km	0.276	0.060	<0.001

*Note*

Thresholds were determined by rejecting the lowest 10% of possible predicted values.

### 3.3 Relative importance of environmental variables in determining species occurrence

**Figure 4:**
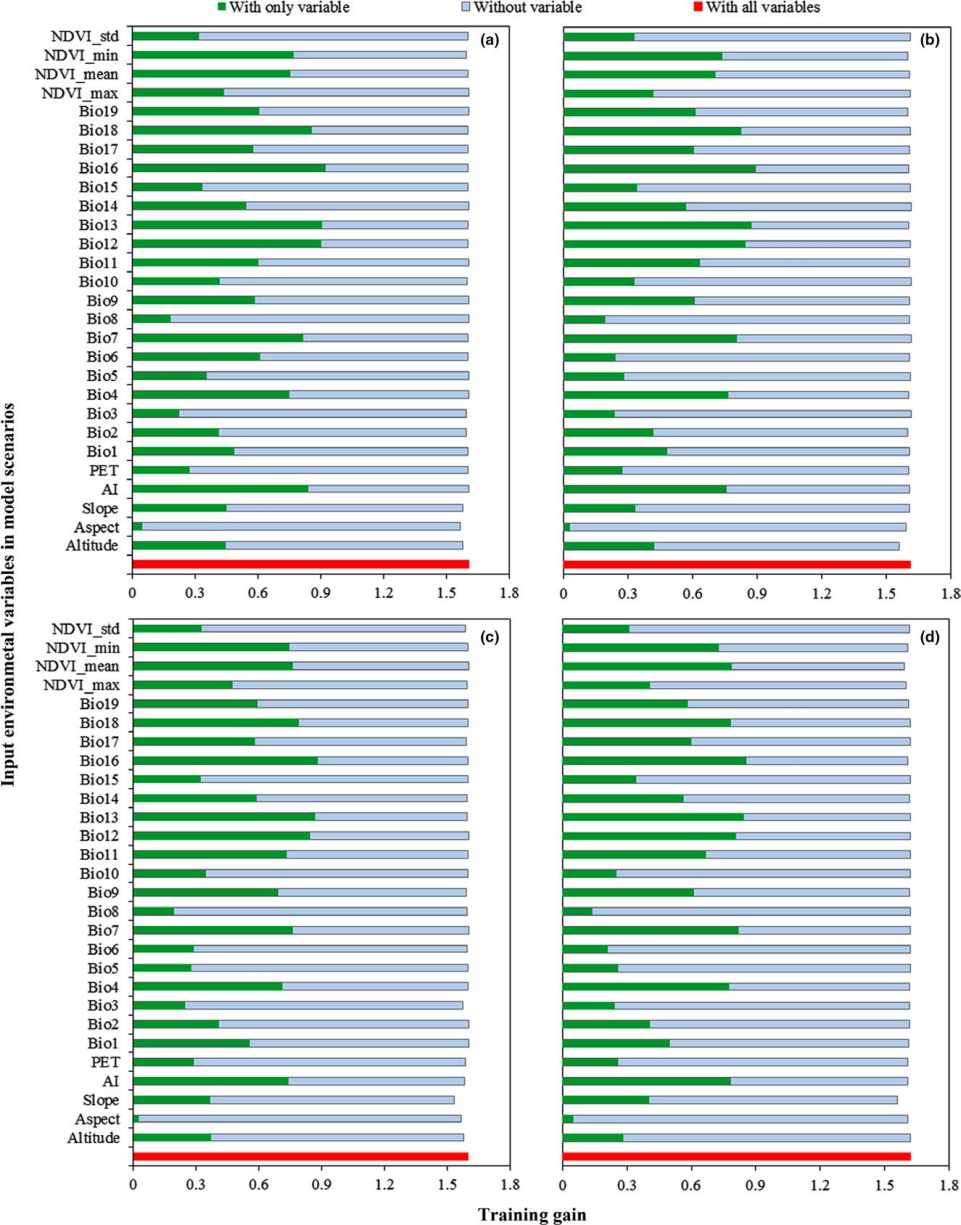
Importance of environmental variables to model the distribution of epiphyllous liverworts from different resolutions: (a) 1 km, (b) 5 km, (c) 10 km, and (d) 15 km. The graphs depict the training gains when a variable is used in isolation, when the variable is excluded, and when all variables are utilized. The gain is a measure of how better the Maxent probability distribution fits the distribution of occurrence data. A variable has useful information when the gain is high as it is used in isolation and has unique information when it reduces the gain most when it is excluded

Jackknife tests were performed to determine key variables influencing epiphyllous liverworts distribution at different spatial scales. The environmental variable with the highest training gain, when used in isolation, is considered to contain the most predictive ability of any variables. The environmental variable reduces the gain the most when it is omitted, which therefore appears to possess the highest amount of information that is not present in the other variables. Figure 4 shows the results of the jackknife experiments, which reveals that the factors that determined the distribution of epiphyllous liverworts for the four resolution models were similar. The total training gain with all variables included for modeling ranged from 1.601 to 1.623, with the gain order of the model scenarios of resolution 15 km > 5 km > 1 km > 10 km. Among the environmental variables involved in the model, the climatic variables including aridity (AI), temperature seasonality (Bio4), temperature annual range (Bio 7), annual precipitation (Bio12), precipitation of wettest month (Bio13), precipitation of wettest quarter (Bio16), and precipitation of warmest quarter (Bio18), and vegetation variables including annual mean NDVI (NDVI_mean) and minimum NDVI (NDVI_min) were among the most important variables contributing to the two models, which possess a training gain of more than 0.7 in all model scenarios. By contrast, topographic variables were not important indicators for a suitable habitat because all training gains were less than 0.5 when the variables were used in isolation, and the gain decreased to less than 0.05 when the variables were omitted (Figure 4).

**Figure 5:**
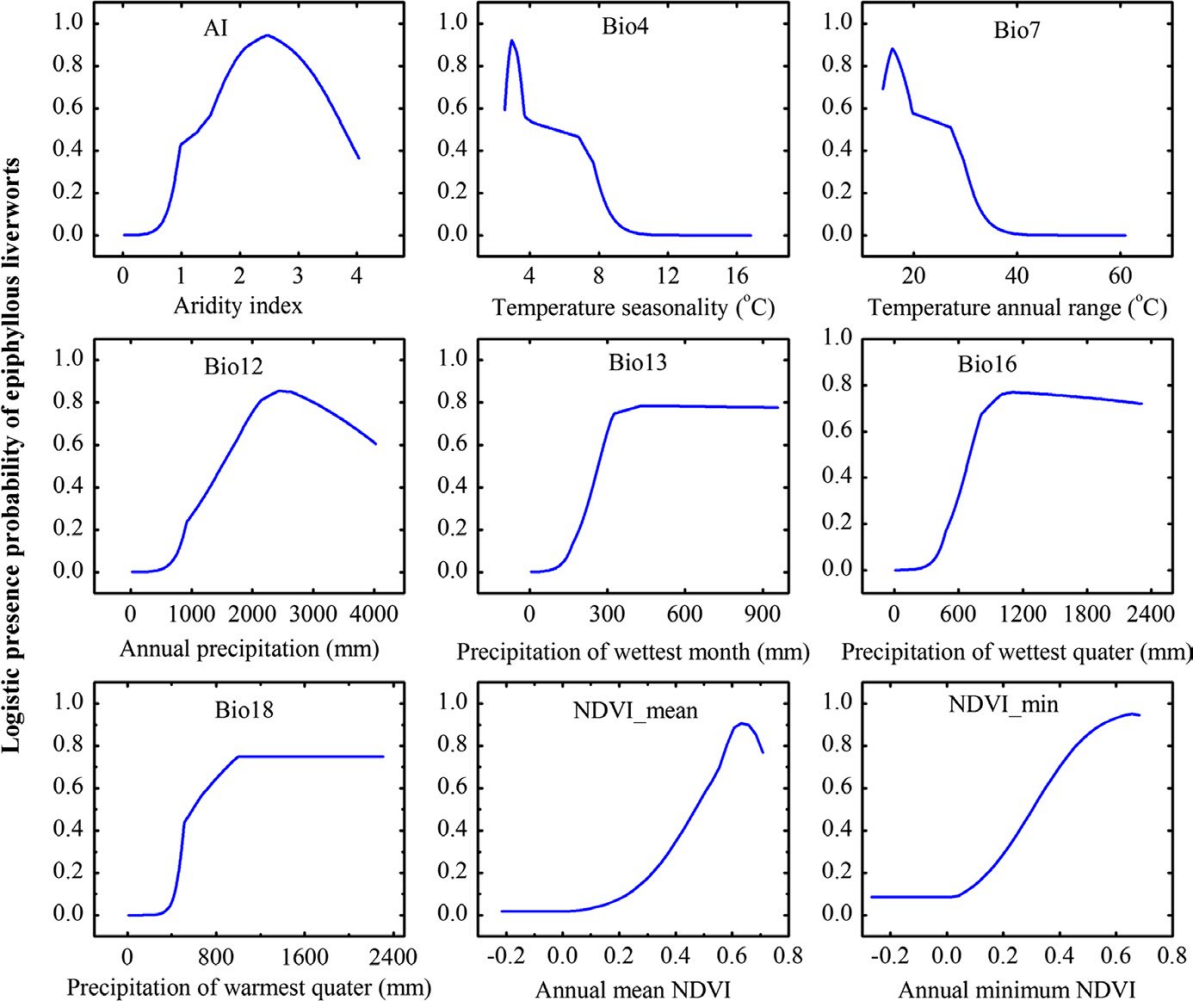
Response curves illustrating the relationship between presence probability of epiphyllous liverworts and environmental variables. These curves show how the response changes for a particular variable used in isolation. The response curves were derived from the 15‐km model in Maxent

Response curves greatly facilitate the interpretation of how environmental factors determine the distribution of a species. The responses of the favorable variables in the prediction for the epiphyllous liverworts in the best performed model, which was 15‐km resolution model, are indicated in Figure 5. According to the response curves, higher values of the AI, Bio4, Bio7, Bio12, Bio13, Bio16, Bio18, NDVI_mean, and NDVI_min were preferable to epiphyllous liverworts presence and only if these variables reached or less than a particular value, epiphyllous liverworts probably occurred. For example, temperature annual range was less than 31°C, annual precipitation was higher than 1000 mm, and annual minimum NDVI exceeded 0.15, and the presence probability of epiphyllous liverworts could reach to 0.2.

## 4 DISCUSSION

### 4.1 Factors determining model performance and distribution range

Spatial scale is a fundamental issue in the construction of the species distribution model. Sampling resolution should optimally be selected to be as coherent with the resolution of the predictor variables and to correspond to the scale relevant for habitat selection (Guisan & Thuiller, 2005). If species records are of vague locations, then a set of predictors available at a fine resolution may need to be aggregated to a coarse resolution (Guisan et al., 2007). Changing the resolution can result in two directions of model performance, that is, slight average toward model degradation at coarse resolution (Guisan et al., 2007) or model improvement at the coarse resolution compared with the fine resolution (Tobalske, 2002). In the present study, model performance exhibited an insignificant trend along resolution coarsening according to AUC and TSS. The effect of the resolution on the model performance could be species‐specific (Gottschalk, Aue, Hotes, & Ekschmitt, 2011; Guisan et al., 2007; Seo, Thorne, Hannah, & Thuiller, 2009). For species in our study, the resolution did not significantly influence model fitting at the regional scale. Nevertheless, 15 km was suggested to be the optimal resolution of all the four resolutions to model epiphyllous liverwort distribution, because it showed the highest model fit and training gain among all model scenarios. Also, the distribution map derived from the 15‐km resolution model showing less suitable area and more fragment distribution patches was more consistent with the observed or real species distribution according to expert knowledge of epiphyllous liverworts and our previous study (Jiang et al., 2014). The higher accuracy achieved by the coarse resolution model indicated that a proper spatial resolution of environmental variables in accordance with the accuracy of occurrence location should be taken into consideration.

As studied by numerous ecologists, sample size is another key issue on the performance of species distribution models (Hernandez, Graham, Master, & Albert, 2006; Stockwell & Peterson, 2002; Wisz et al., 2008). Models with a large number of occurrences in the training set generally performed better and had smaller variances than models built with few occurrences (Guisan et al., 2007). Accurate predictions of species distributions were also based on adequate sampling of environmental variation, because any two geographical regions will differ in the distribution and range of their environmental variation (Graham et al., 2008). Although variability exists across species and between model methods, model accuracy generally decreased with the decrease in sample size. By bad luck, we have not addressed the influence of sample size on the model accuracy, as models of all resolutions were constructed based on the same historical records, in order to detecting the resolution effect directly. Further research is required on this topic, for example, examining changes in model performance by altering sample size. Even so, we are aware of that if the location can be accurately obtained from historical records and sufficient field presence points are observed, then high‐accuracy model performance and species distribution range can be achieved.

### 4.2 Environmental variables accounted for epiphyllous liverworts occurrence at the regional scale

Climate is often considered a predominant range‐determining mechanism at large spatial scale (Blach‐Overgaard, Svenning, Dransfield, Greve, & Balslev, 2010; Guisan et al., 2007; Pearson & Dawson, 2003). The variables most often having the highest contributions in the Maxent model were variables related to precipitation and temperature. For epiphyllous liverworts in China, high annual precipitation and mean temperature increase the presence probability. As we analyzed that AI, Bio13, Bio16, and Bio18 were all closely correlated with annual precipitation, Bio4, and Bio7 were correlated with annual mean temperature tightly. These results reflect that epiphyllous liverworts favor habitats with humid and warm climate, which is consistent with past ecological studies on epiphyllous liverworts (Benavides & Sastre‐De Jesus, 2011; Jiang et al., 2014; Kraichak, 2014; Olarinmoye, 1974). These areas with humid and warm climate determine the geographical distribution of evergreen forests in China, which occur between 18 and 32°N and 98–123°E, within areas dominated by tropical and subtropical climate, with annual mean temperature between 14°C and 26°C, and precipitation ranging from 1,000 to 5,000 mm (Wu, 1980). Annual mean and minimum NDVI also provided meaningful and significant contributions to defining the distribution range and spatial patterns of epiphyllous liverworts. However, the importance of the NDVIs was a bit less than climatic variables in this study, which is inconsistent with our previous study (Jiang et al., 2014). Some reasons could explained: First, it may attributed to the uncertainty of the species occurrence data; second, the long‐time span of historical records may be more sensitive to climate change other than vegetation cover; third, NDVIs originated from the GIMMS (8 km) were of much coarser resolution than that derived from SPOT sensor (1 km). By contrast, topographic variables had an insignificant influence on the regional presence of epiphyllous liverworts, which is consistent with the results of our previous study (Jiang et al., 2014). The descriptions of the known localities demonstrate that epiphyllous liverworts are distributed in a broad range of altitude, from 300 to 2,800 m, and they are sensitive to microclimate and small terrain changes. The topographic effect considerably weakens under a broad scale, with a resolution higher than 1 km.

### 4.3 Importance of historical data

Systematic surveys with constant spatial scale as environmental variables are likely to be more powerful than haphazard historical records in species distribution modeling (Aikio, Duncan, & Hulme, 2010). Historical distributions of organisms in recent and distant (paleontological) past however have provided a platform for assessing biodiversity dynamics with and without anthropogenic influence (Graham et al., 2004). Historical data are considered useful in improving insight into factors that control species distribution, modeling species distribution, predicting the future propagation pattern, and planning long‐term management strategies (Aikio et al., 2010; Kéry, Gardner, & Monnerat, 2010; Wollan, Bakkestuen, Kauserud, Gulden, & Halvorsen, 2008). Existing historical records of epiphyllous liverworts in China represents a time span of nearly 50 years, which is consistent with climatic variables derived from WorldClim reflecting average values of 50 years. These records also cover a wide range across China where predicted high occurrence probability may represent sufficient geographical conditions. Even spatial error exists due to the descriptive localities, which can be reduced after selecting an appropriate spatial resolution of environment layers. Historical data are therefore useful in helping to construct a reliable model when accurate samples are insufficient.

## 5 CONCLUSION

Successfully modeling the past distribution of epiphyllous liverworts based on historical records depended on several factors. Changes in resolution did not significantly affect model fitting performance, but influenced the suitable area and distribution pattern. 15 km was suggested to be the optimal resolution of the four resolutions (1 km, 5 km, 10 km, and 15 km) to model epiphyllous liverwort distribution, because this model possessed the highest model fit and training gain, and more consistent with the real species distribution. Climatic variables, especially humidity‐related variables, such as annual precipitation and aridity, together with vegetation indices contributed significantly in defining species distribution range and spatial patterns. The low predicted area indicates that epiphyllous liverworts only occur in a restricted geographical range in China. The results of our study indicate that epiphyllous liverworts are suitable for the analyses of ecological and biogeographical patterns over time and space, and certainly help in assessing the effect of human disturbance on the distribution and predict future distribution to climate change. The predicted approximate habitat suitability and habitat loss also guide conservation and management.

## CONFLICT OF INTEREST

None declared.

## AUTHOR CONTRIBUTIONS

Yanbin Jiang, Tiejun Wang, and Xiaoming Shao conceived the idea and scope of this manuscript. Yanbin Jiang, Xiaoming Shao, Yupeng Wu, Ronggui Hu, and Ke Huang collected the data. Yanbin Jiang and Tiejun Wang analyzed the results and led the writing of the manuscript. All authors contributed critically to the drafts and gave final approval for publication.

## DATA ACCESSIBILITY

Data available from the Dryad Digital Repository: https://doi.org/10.5061/dryad.2jr1648

## APPENDIX 1

### Historical species records from publications and natural history collections

**Table:**

Province	Site name	Elevation (m)	Survey time	Sources
Anhui	Zhawan, Qimen	200	1982	Wu and Guo (1986)
Fujian	Wuyishan, Guadun	450–1300	1955, 1979–82	Chen and Wu (1964), Wu, Li, and Gao (1983)
	Jiufengshan	400–900	1999	Zhu, Wang, Zhu, and Sun (2001)
	Wanmulin	350–450	1986	Li (1997)
	Nanjing, Shuhaijinshan	400	1963	Zhu and So (1997, 2001)
	Longxishan, Jiangle	1450	1991	Herbarium, Institute of Botany, Chinese Academy of Sciences
Guangdong	Dinghushan	800	1989	Zhu and Wang (1992)
	Heishiding Nature Reserve	350–600	1992	Li (1992)
	Babaoshan	550–1700	1989	Zhu, Hu, and Guo (1992);
	Xinyi		1932	Chen and Wu, (1964)
	Nankunshan, Zengcheng		1932, 53	Chen and Wu (1964)
	Jiulongshan, Lianping	650	1987	Gao and Bi (1988)
Guangxi	Huaping	960	1981	Hu, Jin, and Jin (1981)
	Maoershan Nature Reserve	550	1974	Zhu and So (2001)
	Shiwandashan		1989	Herbarium, Institute of Botany, Chinese Academy of Sciences
	Jiuwandashan	1100	1993	Wang and Jia (1993)
Guizhou	Maolan	420–800	1984	Wu (1988), Zhu and So (2001)
	Fanjingshan	1500–2000	1983	Zhu and So (2001)
	Kuankuoshui	1600	1983	Zhu and So (2001)
	Xiaoqikong	600	1998	Zhu and So (2001)
Hainan	Bawangling Nature Reserve	600–1100	1989	Zhu and So (2001)
	Jianfengling Nature Reserve	320–1200	1941, 62, 84	Wu and Lin, (1994), Zhu and So (2001)
	Diaoluoshan	400–1050	1974, 77, 84	Zhu and So (2001)
	Wuzhishan	650–1200	1977	Zhu and So (2001)
Hongkong	Taimoshan	600–900	1995–97	Zhu and So (2001)
	Taipokau		1996–98	Zhu and So (2001)
	Wukaotang	50	1995–96	Zhu and So (2001)
Hunan	Jinbianxi, Zhangjiajie	460	1992	Zhu and So (2001)
	Mangshan, Yizhang		1974	Zhu and So (2001)
Jiangxi	Jinggangshan	650–950	1984	Li and Wu (1988)
	Wuyishan Nature Reserve	960	1993–94	Ji and Liu (1998b)
	Guanshan Nature Reserve	300–900	1995, 96	Ji, Zheng, Xie, Wu and Qiang (2005)
	Sanqingshan	660	1987	Ji, Liu, Zhang, Chen, and Luo (1999)
	JiulingMufushan, Xiushui	350–400	1994, 95	Ji and Liu (1998a)
	JiulingMufushan, Wuning	300	1994, 95	Ji and Liu (1998a);
	Jiulianshan	450–700	1992, 95	Ji, Xie, Liu, Zhang, and Chen (1998)
Sichuan	Ermeishan	900–1500	1979, 80	Zhu and So (2001)
	Erlangshan	160–1800	1974	Zhu and So (2001)
	Jinfoshan	2100	1984	Zhu and So (2001)
	Moxi		1980	Zhu and So (2001)
	Tiangtang	900–1200	1984	Zhu and So (2001)
Taiwan	Zhibenzhushan		1932	Chen and Wu (1964)
	Taipinghsan		1932	Chen and Wu (1964)
	Alishan		1932	Zhu and So (2001)
	Yuanyanghu, Xinzhu	1670	1998	Zhu and So (2001)
Xizang	Medog	780–2450	1960, 82, 83	Chen and Wu (1964), Wu and Luo (1978)
Yunnan	Daweishan Nature Reserve	1300–1960	1974, 88	Zhu and So (2001)
	Tongbiguan, Longchuan	1100	1974	Zhu and So (2001)
	Huanglianshan		1973	Zhu and So (2001)
	Gongshan, Dulongjiang	1240–2800	1982	Zhu and So (2001)
	Mengyang	850–1200	1936	Chen and Wu (1964)
	Menglun Botanical garden	850–1100	1957, 74, 82	Zhu and So (2001)
	Yiwu	750–1900	1936	Chen and Wu (1964), Zhu and So (2001)
	Mengla	1000	1936, 64, 95	Chen and Wu (1964), Zhu and So (2001)
	Menghai	1300	1936	Chen and Wu (1964)
	Mengzhe	1900	1936	Chen and Wu (1964)
Zhejiang	Wuyanling	600–1140	1987	Zhu and Hu (1991)
	Baishanzu Nature Reserve	600–1200	1990	Zhu, Zhang, and Mao (1992)
	Jiulongshan	400–1600	1981	Liu (1985)
	Fengyangshan	350–1580	1992–1993	Zhu, Ye, and Cai (1994)
	Gutianshan	360	1993	Zhu and So (2001)

## APPENDIX 2

### Threshold determined by the lowest predicted value was associated with any one of the observed presence records (LPT), rejecting the lowest 10% of possible predicted values (T10), and maximum specificity plus sensitivity (Max Se+Sp).

**Table:**

Method	1 km	5 km	10 km	15 km
LPT	0.062	0.097	0.092	0.122
T10	0.210	0.242	0.206	0.276
Max Se+Sp	0.173	0.225	0.125	0.195

## References

[ece34275-bib-0001] Aikio, S. , Duncan, R. P. , & Hulme, P. E. (2010). Herbarium records identify the role of long‐distance spread in the spatial distribution of alien plants in New Zealand. Journal of Biogeography, 37, 1740–1751.

[ece34275-bib-0002] Allouche, O. , Tsoar, A. , & Kadmon, R. (2006). Assessing the accuracy of species distribution models: Prevalence, kappa and the true skill statistic (TSS). Journal of Applied Ecology, 43, 1223–1232.

[ece34275-bib-0003] Austin, M. (2007). Species distribution models and ecological theory: A critical assessment and some possible new approaches. Ecological Modelling, 200, 1–19.

[ece34275-bib-0004] Benavides, J. C. , & Sastre‐De Jesus, I. (2011). Diversity and rarity of epiphyllous bryophytes in a superhumid tropical lowland forest of Chocó‐Colombia. Cryptogamie Bryologie, 32, 119–133.

[ece34275-bib-0005] Blach‐Overgaard, A. , Svenning, J. C. , Dransfield, J. , Greve, M. , & Balslev, H. (2010). Determinants of palm species distributions across Africa: The relative roles of climate, non‐climatic environmental factors, and spatial constraints. Ecography, 33, 380–391.

[ece34275-bib-0006] Braunisch, V. , & Suchant, R. (2010). Predicting species distributions based on incomplete survey data: The trade‐off between precision and scale. Ecography, 33, 826–840.

[ece34275-bib-0007] Butcher, J. A. , Collier, B. A. , Silvy, N. J. , Roberson, J. A. , Mason, C. D. , & Peterson, M. J. (2014). Spatial and temporal patterns of range expansion of white‐winged doves in the USA from 1979 to 2007. Journal of Biogeography, 41, 1947–1956.

[ece34275-bib-0008] Chen, P. C. , & Wu, P. C. (1964). Study on epiphyllous liverworts of China (I). Acta Phytotaxon Sinica, 9, 213–276.

[ece34275-bib-0009] Davison, P. G. (1997). Epiphyllous liverworts newly discovered in the Southern Appalachians. Castanea, 62, 215–218.

[ece34275-bib-0010] Elith, J. , Kearney, M. , & Phillips, S. (2010). The art of modelling range‐shifting species. Methods in Ecology and Evolution, 1, 330–342.

[ece34275-bib-0011] Engler, R. , Guisan, A. , & Rechsteiner, L. (2004). An improved approach for predicting the distribution of rare and endangered species from occurrence and pseudo‐absence data. Journal of Applied Ecology, 41, 263–274.

[ece34275-bib-0012] Gao, C. , & Bi, P. (1988). Epiphyllous liverworts of Daiwu Shan, Jiulong (Kowloon). Acta Botanica Yunnanica, 10, 353–356.

[ece34275-bib-0013] Gottschalk, T. K. , Aue, B. , Hotes, S. , & Ekschmitt, K. (2011). Influence of grain size on species‐habitat models. Ecological Modelling, 222, 3403–3412.

[ece34275-bib-0014] Graham, C. H. , Ferrier, S. , Huettman, F. , Moritz, C. , & Peterson, A. T. (2004). New developments in museum‐based informatics and applications in biodiversity analysis. Trends in Ecology & Evolution, 19, 497–503. 16701313 10.1016/j.tree.2004.07.006

[ece34275-bib-0501] Graham, C. H. , Elith, J. , Hijmans, R. J. , Guisan, A. , Peterson, A. T. , Loiselle, B. A. , & Gro, N. P. S. W. (2008). The influence of spatial errors in species occurrence data used in distribution models. Journal of Applied Ecology, 45, 239–247.

[ece34275-bib-0015] Guisan, A. , Graham, C. H. , Elith, J. , & Huettmann, F. (2007). Sensitivity of predictive species distribution models to change in grain size. Diversity and Distributions, 13, 332–340.

[ece34275-bib-0016] Guisan, A. , & Thuiller, W. (2005). Predicting species distribution: Offering more than simple habitat models. Ecology Letters, 8, 993–1009. 10.1111/j.1461-0248.2005.00792.x 34517687

[ece34275-bib-0017] Guisan, A. , & Zimmermann, N. E. (2000). Predictive habitat distribution models in ecology. Ecological Modelling, 135, 147–186.

[ece34275-bib-0018] Hernandez, P. A. , Graham, C. H. , Master, L. L. , & Albert, D. L. (2006). The effect of sample size and species characteristics on performance of different species distribution modeling methods. Ecography, 29, 773–785.

[ece34275-bib-0502] Hijmans, R. J. , Cameron, S. E. , Parra, J. L. , Jones, P. G. , & Jarvis, A. (2005). Very high resolution interpolated climate surfaces for global land areas. International Journal of Climatology, 25, 1965–1978.

[ece34275-bib-0019] Hu, S. , Jin, J. , & Jin, D. (1981). A preliminary investigation on the distribution of bryophytes in broadleaved evergreen forest in Huaping, Guangxi. Guihaia, 1, 1–8.

[ece34275-bib-0020] Jackson, C. R. , & Robertson, M. P. (2011). Predicting the potential distribution of an endangered cryptic subterranean mammal from few occurrence records. Journal for Nature Conservation, 19, 87–94.

[ece34275-bib-0021] Ji, M.‐C. , & Liu, Z.‐L. (1998a). A preliminary report on the epiphyllous liverworts from Jiuling Mufu Mountain of Jiangxi Province, China. Journal of Natural Museum, 16, 13–16.

[ece34275-bib-0022] Ji, M. , & Liu, Z. (1998b). A preliminary report on epiphyllous liverworts in Wuyishan Nature Reserve of Jiangxi Province. Acta Agriculturae Universitatis Jiangxiensis 20.

[ece34275-bib-0023] Ji, M.‐C. , Liu, Z.‐L. , Zhang, Z.‐Y. , Chen, Y.‐J. , & Luo, L.‐C. (1999). A preliminary report on the epiphyllous liverworts species from Jiangxi Province, China. Jiangxi Science, 17, 39–41.

[ece34275-bib-0024] Ji, M. , Xie, Q. , Liu, Z. , Zhang, Z. , & Chen, Y. (1998). Study on the epiphyllous liverworts from Jiulianshan Nature Reserve of Jiangxi Province, China. Journal of Wuhan Botanical Research, 16, 33–38.

[ece34275-bib-0025] Ji, M. , Zheng, G. , Xie, Y. , Wu, H. , & Qiang, S. (2005). Epiphyllous liverworts from Guanshan Nature Reserve of Jiangxi Province. Journal of Zhejiang Forestry College, 22, 370–374.

[ece34275-bib-0026] Jiang, Y. , Wang, T. , de Bie, C. A. J. M. , Skidmore, A. K. , Liu, X. , Song, S. , … Shao, X. (2014). Satellite‐derived vegetation indices contribute significantly to the prediction of epiphyllous liverworts. Ecological Indicators, 38, 72–80.

[ece34275-bib-0027] Johnson, S. A. , Ober, H. K. , & Adams, D. C. (2017). Are keystone species effective umbrellas for habitat conservation? A spatially explicit approach. Journal for Nature Conservation, 37, 47–55.

[ece34275-bib-0028] Kamimura, M. (1939). Studies on the epiphyllous hepaticae and its attached plants in Sikoku, Japan. Japanese Journal of Botany, 15, 63–83.

[ece34275-bib-0029] Kéry, M. , Gardner, B. , & Monnerat, C. (2010). Predicting species distributions from checklist data using site‐occupancy models. Journal of Biogeography, 37, 1851–1862.

[ece34275-bib-0030] Kraichak, E. (2014). Microclimate Fluctuation Correlated with Beta Diversity of Epiphyllous Bryophyte Communities. Biotropica, 46, 575–582.

[ece34275-bib-0031] Li, Z. (1992). Studies on epiphyllous liverworts in China (V). Epiphyllous liverworts in Heishiding Nature Reserve, Guangdong Province. Botanical Journal of South China, 1, 23–27.

[ece34275-bib-0032] Li, D. (1997). A study on epiphyllous liverworts of Wanmulin Nature Reserve in Fujian Province, E China. Chenia, 3–4, 63–68.

[ece34275-bib-0033] Li, D. , & Wu, P. (1988). A study of the epiphyllous liverworts of China (IV), the epiphyllous liverworts on Jinggangshan, Jiangxi Province. Inversigatio et Studium Naturae, 8, 38–42.

[ece34275-bib-0034] Liu, Z. (1985). A preliminary study of Hepaticae from Mt. Jiulongshan Prov. Zhejiang, Eastern China. Investigatio et Studium, 5, 133–152.

[ece34275-bib-0035] Luo, J.‐S. (1990). A synopsis of Chinese epiphyllous liverworts. Tropical Bryology, 2, 161–166.

[ece34275-bib-0036] Moya, W. , Jacome, G. , & Yoo, C. (2017). Past, current, and future trends of red spiny lobster based on PCA with MaxEnt model in Galapagos Islands, Ecuador. Ecology and Evolution, 7, 4881–4890. 28690816 10.1002/ece3.3054 PMC5496532

[ece34275-bib-0037] Ning, S. Y. , Wei, J. F. , & Feng, J. N. (2017). Predicting the current potential and future world wide distribution of the onion maggot, Delia antiqua using maximum entropy ecological niche modeling. PLoS ONE, 12(2), e0171190. 28158259 10.1371/journal.pone.0171190 PMC5291381

[ece34275-bib-0038] Olarinmoye, S. O. (1974). Ecology of epiphyllous liverworts: Growth in 3 natural habitats in western Nigeria. Journal of Bryology, 8, 275–289.

[ece34275-bib-0039] Pearce, J. , & Ferrier, S. (2000). Evaluating the predictive performance of habitat models developed using logistic regression. Ecological Modelling, 133, 225–245.

[ece34275-bib-0040] Pearson, R. G. , & Dawson, T. P. (2003). Predicting the impacts of climate change on the distribution of species: Are bioclimate envelope models useful?. Global Ecology and Biogeography : A Journal of Macroecology, 12, 361–371.

[ece34275-bib-0041] Pearson, R. G. , Raxworthy, C. J. , Nakamura, M. , & Peterson, A. T. (2007). Predicting species distributions from small numbers of occurrence records: A test case using cryptic geckos in Madagascar. Journal of Biogeography, 34, 102–117.

[ece34275-bib-0042] Peng, D. , Liu, S. X. , & Wu, P. C. (2002). Studies on the epiphyllous liverworts of China VIII – The epiphyllous liverworts of Houhe national nature reserve. Journal of Wuhan Botanical Research, 20, 199–201.

[ece34275-bib-0043] Phillips, S. J. , Anderson, R. P. , & Schapire, R. E. (2006). Maximum entropy modeling of species geographic distributions. Ecological Modelling, 190, 231–259.

[ece34275-bib-0044] Phillips, S. J. , & Dudik, M. (2008). Modeling of species distributions with Maxent: New extensions and a comprehensive evaluation. Ecography, 31, 161–175.

[ece34275-bib-0045] Pócs, T. (1982). An epiphyllous liverwort community from the Caucasus Mountains. Bryologische Beiträge, 1, 13–22.

[ece34275-bib-0046] Pócs, T. (1996). Epiphyllous liverworts diversity at worldwide level and its threat and conservation. Anales del Instituto de Biología de la Universidad Nacional Autónoma de México Seris Botanica, 67, 109–127.

[ece34275-bib-0047] Porley, R. D. (1996). Foliicolous Metzgeria fruticulosa on box leaves in the Chiltern Hills, England. Journal of Bryology, 19, 188–189.

[ece34275-bib-0048] Prates‐Clark, C. D. , Saatchi, S. S. , & Agosti, D. (2008). Predicting geographical distribution models of high‐value timber trees in the Amazon basin using remotely sensed data. Ecological Modelling, 211, 309–323.

[ece34275-bib-0049] Risk, A. C. , Richardson, C. , & Davison, P. (2011). Epiphyllous bryophytes in the Appalachian Plateau of Kentucky and Tennessee, U.S.A. Bryologist, 114, 289–297.

[ece34275-bib-0050] Rosenzweig, M. L. (1995). Species diversity in space and time. Cambridge, UK: Cambridge University Press.

[ece34275-bib-0051] Saatchi, S. , Buermann, W. , Ter Steege, H. , Mori, S. , & Smith, T. B. (2008). Modeling distribution of Amazonian tree species and diversity using remote sensing measurements. Remote Sensing of Environment, 112, 2000–2017.

[ece34275-bib-0052] Schuster, R. M. (1959). Epiphyllous hepaticae in the Southern Appalachians. The Bryologist, 62, 52–55.

[ece34275-bib-0053] Seo, C. , Thorne, J. H. , Hannah, L. , & Thuiller, W. (2009). Scale effects in species distribution models: Implications for conservation planning under climate change. Biology Letters, 5, 39–43. 18986960 10.1098/rsbl.2008.0476 PMC2657743

[ece34275-bib-0054] Sérgio, C. , Figueira, R. , Draper, D. , Menezes, R. , & Sousa, A. J. (2007). Modelling bryophyte distribution based on ecological information for extent of occurrence assessment. Biological Conservation, 135, 341–351.

[ece34275-bib-0055] Shirasaki, H. (1997). Distribution and ecology of the epiphyllous liverwort Cololejeunea nakajimae in the winter snow‐covered district of Niigata Prefecture and its adjacent regions, central Japan. Bryological Research, 7, 1–7.

[ece34275-bib-0056] Sjögren, E. (1975). Epiphyllous bryophytes from Maderia. Botanisk Tidskrift, 69, 217–288.

[ece34275-bib-0057] Sjögren, E. (1997). Epiphyllous bryophytes in the Azores Islands. Arquipélago Life and Marine Sciences, 15A, 1–49.

[ece34275-bib-0058] Stockwell, D. R. B. , & Peterson, A. T. (2002). Effects of sample size on accuracy of species distribution models. Ecological Modelling, 148, 1–13.

[ece34275-bib-0059] Tobalske, C. (2002). Effects of spatial scale on the predictive ability of habitat models for the green woodpecker in Switzerland In Scott M. J., Heglund P. J., Morrison M. L., Haufler J. B., Raphael M. G., Wall W. A., & Samson F. B. (Eds.), Predicting species occurrences: Issues of accuracy and scale (pp. 197–204). Washington, DC: Island Press.

[ece34275-bib-0060] Vitt, D. H. , Ostafichuk, M. , & Brodo, I. M. (1973). Foliicolous bryophytes and lichens of Thuja plicata in western British Columbia. Canadian Journal of Botany, 51, 571–580.

[ece34275-bib-0061] Wang, M. Z. , & Jia, Y. (1993). A preliminary study of Hepaticae from Mt. Jiuwan of Guangxi, South China. Chenia, 1, 125–131.

[ece34275-bib-0062] Warren, D. L. , & Seifert, S. N. (2011). Ecological niche modeling in Maxent: The importance of model complexity and the performance of model selection criteria. Ecological Applications, 21, 335–342. 21563566 10.1890/10-1171.1

[ece34275-bib-0063] Wisz, M. S. , Hijmans, R. J. , Li, J. , Peterson, A. T. , Graham, C. H. , Guisan, A. , & Distribut, N. P. S. (2008). Effects of sample size on the performance of species distribution models. Diversity and Distributions, 14, 763–773.

[ece34275-bib-0064] Wollan, A. K. , Bakkestuen, V. , Kauserud, H. , Gulden, G. , & Halvorsen, R. (2008). Modelling and predicting fungal distribution patterns using herbarium data. Journal of Biogeography, 35, 2298–2310.

[ece34275-bib-0065] Wu, Z. Y. (1980). Vegetation of China. Beijing, China: Science Press.

[ece34275-bib-0066] Wu, P. (1988). The epiphyllous liverworts in Maolan, Libo County, SW China. Guihaia, 6, 335–338.

[ece34275-bib-0067] Wu, P. C. , & Guo, X. H. (1986). A report on the epiphyllous liverworts in Anhui province, China. Acta Phytotaxon Sinica, 24, 136–138.

[ece34275-bib-0068] Wu, P. , Li, D. , & Gao, C. (1983). Studies on the epiphyllous liverworts of China (II), the epiphyllous liverworts on Wuyi MT., Fujian Province. Wuyi Science Journal, 9, 1–6.

[ece34275-bib-0069] Wu, P. , & Lin, P. (1994). Studies on the epiphyllous liverworts of China VI. The epiphyllous liverworts of Hainan Island. Chenia, 2, 115–120.

[ece34275-bib-0070] Wu, P. C. , & Luo, J. X. (1978). Studies on the epiphyllous liverworts of China (II) –The epiphyllous liverworts from Tibet. Acta Botanica Sinica, 16, 102–112.

[ece34275-bib-0071] Zhu, R. , & Hu, R. (1991). A study on the epiphyllous liverworts from Wuyanling of Zhejiang Province. Journal of East China Normal University (Natural Science), 1991, 98–103.

[ece34275-bib-0072] Zhu, R.‐L. , Hu, R. L. , & Guo, X.‐H. (1992). A study on epiphyllous liverworts from Babaoshan, Guangdong. Acta Botanica Yunnanica, 14, 264–268.

[ece34275-bib-0073] Zhu, R. L. , & So, M. L. (1997). A new record of the genus Otolejeunea (Hepaticae, Lejeuneaceae) in subtropical China. Annales Botanici Fennici, 34, 285–289.

[ece34275-bib-0074] Zhu, R. L. , & So, M. L. (2001). Epiphyllous liverworts of China. Nova Hedwigia Beiheft, 121, 1–418.

[ece34275-bib-0075] Zhu, R. , & Wang, Y. (1992). A preliminary revision of epiphyllous liverworts from Dinghushan. Journal of East China Normal University (Natural Science), 1992, 90–97.

[ece34275-bib-0076] Zhu, J. , Wang, Y. , Zhu, R. , & Sun, S. (2001). Epiphyllous liverworts of the eastern Jiufeng Mountain, Fujian Province. Journal of East China Normal University (Natural Science), 4, 96–102.

[ece34275-bib-0077] Zhu, R. L. , Ye, L. X. , & Cai, H. Z. (1994). Epiphyllous liverworts of Fengyangshan Nature Reserve, Zhejiang Province, China. Bryologist, 97, 277–279.

[ece34275-bib-0078] Zhu, R. , Zhang, G. , & Mao, X. (1992). Resources of epiphyllous liverworts in Baishanzu Nature Reserve of Zhejiang Province. Journal of Plant Resources and Environment, 1, 19–23.

